# Fractionation of Phenolic Compounds Extracted from Propolis and Their Activity in the Yeast *Saccharomyces cerevisiae*

**DOI:** 10.1371/journal.pone.0056104

**Published:** 2013-02-07

**Authors:** Tanja Petelinc, Tomaž Polak, Lea Demšar, Polona Jamnik

**Affiliations:** Department of Food Science and Technology, Biotechnical Faculty, University of Ljubljana, Ljubljana, Slovenia; Instituto de Salud Carlos III, Spain

## Abstract

We have here investigated the activities of Slovenian propolis extracts in the yeast *Saccharomyces cerevisiae*, and identified the phenolic compounds that appear to contribute to these activities. We correlated changes in intracellular oxidation and cellular metabolic energy in these yeasts with the individual fractions of the propolis extracts obtained following solid-phase extraction. The most effective fraction was further investigated according to its phenolic compounds.

## Introduction

Propolis is a resinous substance that is collected from certain plants by bees. The bees use it as a sealer in their hives and to prevent the decomposition of creatures that invade the hive and the bees can kill, but cannot remove. Although the composition of propolis varies, depending on the place and time of its collection [1], in general it contains resins and balsams (50%), waxes (30%), aromatic and essential oils (10%), pollen (5%) and other organic matter (5%) [2], [3]. Propolis has a broad spectrum of biological activities, including antioxidative, anti-inflammatory, immunomodulatory, anticancer, antibacterial, antiviral, antifungal and antiparasitic effects [4], [5]. Although propolis is a mixture of compounds, its pharmacological activities are reported to arise from its flavonoids and phenolic acids, and their esters [6].

In the present study, the activity of propolis was investigated using stationary phase *Saccharomyces cerevisiae* yeast as the model organism. In this system in our previous study, we showed that propolis decreases intracellular oxidation, with its antioxidative activity in yeast arising from only a part of it [7]. To better understand this antioxidative activity of propolis in yeast, we have here further investigated the activities of the phenolic compounds of propolis. Therefore, the objectives of this study were to: (1) fractionate a crude propolis extract using polarity-based solid-phase extraction; (2) determine any correlations between the total phenolic content of individual propolis fractions and its antioxidant activity *in vitro*/*in vivo*; (3) determine any correlations between the total phenolic content of individual fractions of propolis and the cellular metabolic energy; and (4) identify the phenolic compounds of any fractions that promote changes in intracellular oxidation and cellular metabolic energy in yeast.

## Materials and Methods

### Ethics Statement

No specific permits were required for the described field studies. The location is not privately-owned or protected in any way. The field studies did not involve endangered or protected species.

### Chemicals and Standards

Folin-Ciocalteu’s reagent, 96% ethanol, glucose, methanol, formic acid, sodium carbonate and acetonitrile were from Merck. The 2,2-diprenyl-1-picryhydrazyl radical (DPPH), gallic acid and 2′,7′-dichlorodihydrofluorescein diacetate were from Sigma. Yeast extract and peptone were from Biolife. Ammonium formate was from Fluka.

### Sample Preparation

Propolis was collected from bee hive in the Savinjska Valley in Slovenia during the autumn of 2010. The propolis (10 g) was extracted with 70% ethanol (100 mL) by mixing for 1 h at room temperature. The crude extract was recovered by centrifugation (3000 *g*, 5 min) and concentrated under vacuum using a rotary evaporator.

### Solid-phase Extraction

**Figure 1:**
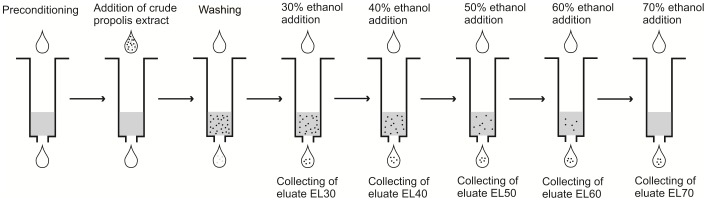
Preparation of the EL30 to EL70 eluates from the crude propolis extract by solid-phase extraction.

Solid-phase extraction (SPE) was used to clean the crude propolis extract, whereby it was separated into five elution fractions according to polarity. Crude propolis extract (200 µL) was mixed with 20 mM ammonium formate (200 µL), and then added to a Strata-X (33 u Polymeric Reversed Phase 60 mg/3 mL 8B-S100-UBJ) SPE cartridge (Phenomenex) that had previously been conditioned with methanol (2 mL) and equilibrated with 20 mM ammonium formate, pH 3.2 (2 mL). After the loading of the sample, the cartridge was washed with 20 mM ammonium formate in 15% methanol (2 mL), and vacuum-dried for 3 min. For cleaning of the crude propolis extract, a cartridge was eluted with 96% ethanol (2 mL), to obtain the cleaned propolis ethanolic extract (EE96). For separation of the crude propolis extract, a cartridge was eluted with 30% ethanol (2 mL), followed by 40% ethanol (2 mL), 50% ethanol (2 mL), 60% ethanol (2 mL), 70% ethanol (2 mL) (Fig. 1). This thus provided the propolis ethanol eluates, as the 30% (EL30) to 70% (EL70) ethanol eluates for further analysis.

### Determination of Total Phenolic Content

The total phenolic contents of the cleaned propolis EE96 and the eluates (EL30–EL70) were determined using the Folin-Ciocalteu method [8]. Each sample (50 µL) was mixed with distilled water (700 µL) and Folin-Ciocalteu reagent (125 µL). After 5 min, 20% (w/v) Na_2_CO_3_ (125 µL) was added and the samples were mixed. Absorbance was measured after 90 min incubation in the dark at room temperature, using a Safire II microplate reader (Tecan) (λ = 765 nm). The data expressed as g gallic acid equivalents (GAE)/L were estimated from the calibration curve using gallic acid as standard (absorbance at 765 nm).

### Determination of DPPH Free Radical Scavenging Activity

The *in vitro* antioxidative activities of the cleaned propolis EE96 and the eluates (EL30–EL70) were evaluated using the 2,2-diphenyl-1-picryl-hydrazyl (DPPH) free radical scavenging method [9]. Each sample (25 µL) was mixed with distilled water (225 µL) and DPPH solution (0.05 mg/L in 96% ethanol; 1 mL). The absorbance was measured after 30 min incubation in the dark at room temperature, using a Safire II microplate reader (Tecan) (λ = 517 nm). The data expressed as g GAE/L were estimated from the calibration curve using gallic acid as standard (absorbance at 517 nm).

### Yeast Strain and Cultivation

The yeast *Saccharomyces cerevisiae* ZIM 2155 was obtained from the Culture Collection of Industrial Microorganisms (ZIM) of the Biotechnical Faculty, University of Ljubljana, Ljubljana, Slovenia.

The yeast were cultivated in yeast extract (10 g/L), peptone (20 g/L), glucose (20 g/L) (YEPD) medium at 28°C and with agitation at 220 rpm, until their stationary phase. The yeast were then centrifuged at 4000 *g* for 3 min, washed once with phosphate-buffered saline (PBS) and resuspended in PBS at 1×10^8^ cells/mL. The yeast was further incubated at 28°C and 220 rpm, for 96 h.

### Yeast Treatment

Following the 96 h of incubation in PBS, the yeasts were treated with either the cleaned propolis EE96 or with the particular eluates (EL30–EL70). After a further 2 h of incubation at 28°C and 220 rpm, samples were taken for the determination of: (1) intracellular oxidation; (2) cellular metabolic energy; (3) cell viability; and (4) cellular uptake of individual phenolic compounds.

### Determination of Intracellular Oxidation

Intracellular oxidation was estimated using 2′,7′-dichlorodihydrofluorescein (H_2_DCF), which reacts with oxidants, thus revealing the presence of reactive oxygen species (ROS). This was given to the yeast as H_2_DCF diacetate, which penetrates the plasma membrane and is hydrolyzed inside cells by nonspecific esterases. The nonfluorescent H_2_DCF can then be oxidized to fluorescent 2′,7′-DCF, the levels of which are measured fluorimetrically [10].

After 2 h of treatment, the yeast from 2 mL cell suspensions were sedimented by centrifugation (14,000 *g*, 5 min), and washed three times with 50 mM potassium phosphate buffer (pH 7.8). The cell pellets were resuspended in 9 volumes of 50 mM potassium phosphate buffer (to 10%, v/v) and incubated at 28°C for 5 min. The ROS-sensing dye H_2_DCF diacetate was added from a 1 mM stock solution in 96% ethanol, to a final concentration of 10 µM. After a 20 min incubation at 28°C and 220 rpm, the fluorescence of the yeast suspension was measured, using the kinetic mode of a Safire II microplate reader (Tecan). The excitation and emission wavelengths of DCF were 488 nm and 520 nm, respectively. The data are expressed as fluorescence relative to control.

### Determination of Cellular Metabolic Energy

The cellular metabolic energy was determined using the BacTiter-Glo™ Microbial Cell Viability Assay (Promega), according to the manufacturer’s instructions. After 2 h of treatment, 100 µL cell suspension (washed three times with filtered PBS) at 1×10^7^ cells/mL, and 100 µL BacTiter-Glo™ reagent were placed in 96-well microplates and mixed. After 5 min the luminescence was measured using a Safire II microplate reader (Tecan). The data are expressed as luminescence relative to control.

### Cell Viability Determination

Cell viability was measured as the cell membrane integrity, using LIVE/DEAD® Funga Light TM Yeast Viability kits (Molecular Probes), according to the manufacturer’s instructions. After 2 h of treatment, the yeast from 1 mL cell suspension were sedimented by centrifugation (14,000 *g*, 5 min), and washed three times with filtered PBS. A cell suspension in filtered PBS at 1×10^6^ cells/mL was prepared. Then 1 µL SYTO® 9 and 1 µL propidium iodide were added in the dark to 1 mL of cell suspension, and the samples were vortexed and incubated at 37°C for 30 min, with vortexing every 10 min. After the incubations, the fluorescence was measured using a Safire II microplate reader (Tecan). The excitation/emission wavelengths for these two dyes are 480/500 nm for SYTO® 9, and 490/635 nm for propidium iodide. The data are expressed as fluorescence normalized to OD_650_ relative to the control.

### Determination of Cellular Uptake of Phenolic Compounds

To study the cellular uptake of the phenolic compounds, the phenolic profile was determined in the PBS buffer immediately after the addition of 1% eluates (i.e. before yeast exposure) and after 2 h of exposure of yeast cells to eluates. The samples were centrifuged (4000 *g*, 3 min) and the supernatants obtained were first cleaned using SPE (according to procedure described for EE96) and then analyzed using LC-DAD, to obtain the phenolic profile.

### Liquid Chromatography-diode Array Detection Analysis

The samples were diluted 20-fold in 1% formic acid in 50% methanol and analyzed by liquid chromatography-diode array detection (LC-DAD). The LC system consisted of an Agilent 1100 model G1312A binary pump and a model G1330B autosampler (Agilent Technologies). This reversed-phase HPLC separation was carried out using a Gemini C18 column (150 mm×2.0 mm internal diameter; 3 µm particle size), which was protected by a Gemini C18 security guard cartridge (4.0 mm×2.0 mm internal diameter) (Phenomenex). The mobile phase comprised aqueous 1% formic acid (A) and acetonitrile (B), and the following gradient was used: 0–5 min: 10% B; 5–50 min: 10%–60% B; 50–52 min: 60%–80% B; 52–60 min: 80% B; 60–70 min: 80%–10% B; 70–80 min: 10% B. The column was maintained at 25°C, with an injection volume of 20 µL and a flow rate of 0.2 mL/min.

### Liquid Chromatography-mass Spectrometry Analysis

To identify the phenolic compounds, liquid chromatography-mass spectrometry (LC-MS) was used. The samples were diluted 20-fold in 1% formic acid in 50% methanol and analyzed by LC-MS. A Micromass Quattro Micro mass spectrometer equipped with an electrospray ionization source was operated in negative ion mode (Waters, Milford, MA, USA). The mass spectra were recorded with the following operating parameters: capillary voltage, 3.0 kV; cone voltage, 25 V; extractor, 5 V. The source temperature was 100°C, and the desolvation temperature was 350°C. The cone gas flow was set at 50 L/h, while the desolvation gas flow was set to 400 L/h, and the collision energy at 20 V. The phenolic compounds were identified on the basis of m/z of the [M-H]^−^ and MS^2^ ions.

### Statistical Analysis

The data are expressed as means ±S.D., as determined from triplicate analysis. Duncan’s multiple range tests, at P<0.05, determined the significant differences among the means.

## Results and Discussion

**Figure 2:**
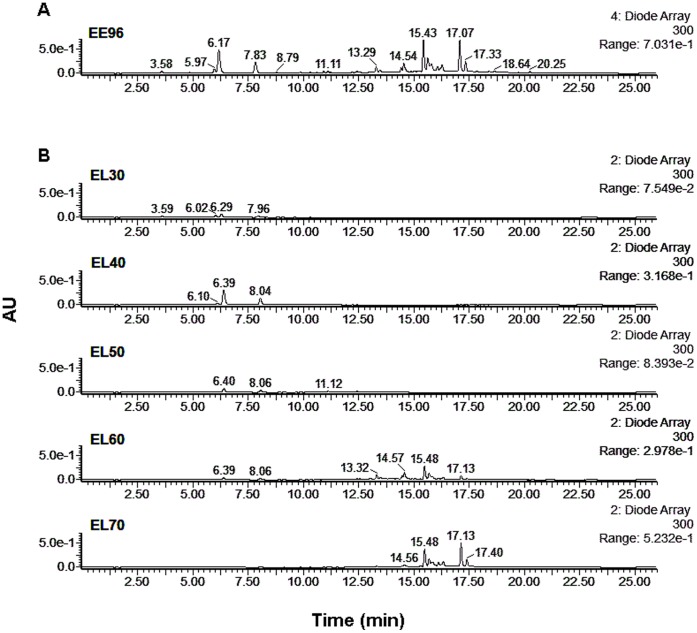
LC-DAD (300 nm) chromatogram of the phenolic compounds following solid-phase extraction without fractionation (A: EE96) and with fractionation (B: EL30–EL70). AU, arbitrary units.

The present study is a continuation of our previous study [7], where we showed that with propolis, the antioxidative activity is not related to the whole propolis extract, but to only a part of it. To better understand which phenolic compounds are responsible for the antioxidative activity of propolis in cells, here fractionation of phenolic compounds in a crude ethanolic propolis extract was performed using SPE (Fig. 1). Five sequential eluates that varied in polarity were obtained, according to the ethanol used for the elution (30% to 70%): EL30, EL40, EL50, EL60 and EL70, from more to less polar, respectively. For the cleaned ethanolic extract of propolis (EE96) as well for the EL30 to EL70 eluates, the phenolic profiles were determined using LC-DAD (Fig. 2). As expected, these profiles differed between the eluates.

Here, as well as the phenolic profile, the total phenolic content and free radical scavenging activity of EE96 and EL30 to EL70 were determined (Table 1). The highest total phenolic content was seen for eluates EL60 and EL70 (0.82 g GAE/L and 0.83 g GAE/L, respectively), which also showed the highest *in vitro* antioxidative activities, measured as the free radical scavenging activity (1.20 g GAE/L and 1.22 g GAE/L, respectively). Thus, eluates EL30 to EL50 showed lower total phenolic contents (0.26 g GAE/L to 0.39 g GAE/L), which was reflected in their lower *in vitro* antioxidative activities (0.18 g GAE/L to 0.33 g GAE/L). The sum of the total phenolic content in these eluates was higher compared to the total phenolic content of EE96. This was expected, as the total volume of ethanol used for elution of the eluates (EL30 to EL70) was five-fold higher than the volume of ethanol used for elution of the cleaned propolis EE96 (Fig. 1). Therefore, some of the phenolic compounds from the crude propolis extract were lost in the process of obtaining EE96.

**Table 1:** Total phenolic content and evaluation of the free radical scavenging activity of EE96 and eluates EL30–EL70.

Eluate	Total phenolic content (g GAE/L)	Free radical scavenging activity (g GAE/L)
EE96	1.99±0.01^a^	1.88±0.01^a^
EL30	0.26±0.01^d^	0.18±0.08^d^
EL40	0.39±0.01^c^	0.33±0.08^c^
EL50	0.26±0.01^d^	0.30±0.05^c^
EL60	0.82±0.01^b^	1.20±0.03^b^
EL70	0.83±0.02^b^	1.22±0.02^b^

Data are means ±S.D. (n = 3); values in the same column followed by the same letter (a-d) are not statistically different (P<0.05), as measured by Duncan’s test.

EE96, ethanolic extract of crude propolis extract prepared using SPE with 96% ethanol elution.

EL30-EL70, eluates of the crude propolis extract prepared using SPE with 30% to 70% ethanol elution.

GAE, gallic acid equivalent.

Fractionation of these compounds extracted from propolis has been reported previously [11], [12], where nanofiltration [11] and supercritical fluid extraction [12] were used. In the case of nanofiltration, the compounds from an ethanolic extract of propolis were fractionated according to their molecular weights. The DPPH test was performed, and it was shown that the antioxidative activity was proportional to the flavonoid content of the fractions. The same was concluded for the fractions obtained using supercritical fluid extraction.

On the bases of *in vitro* studies, predictions about the activities of the phenolic compounds in the cell are usually done. However, this extrapolation can be misleading and additional *in vivo* studies need to be conducted.

**Figure 3:**
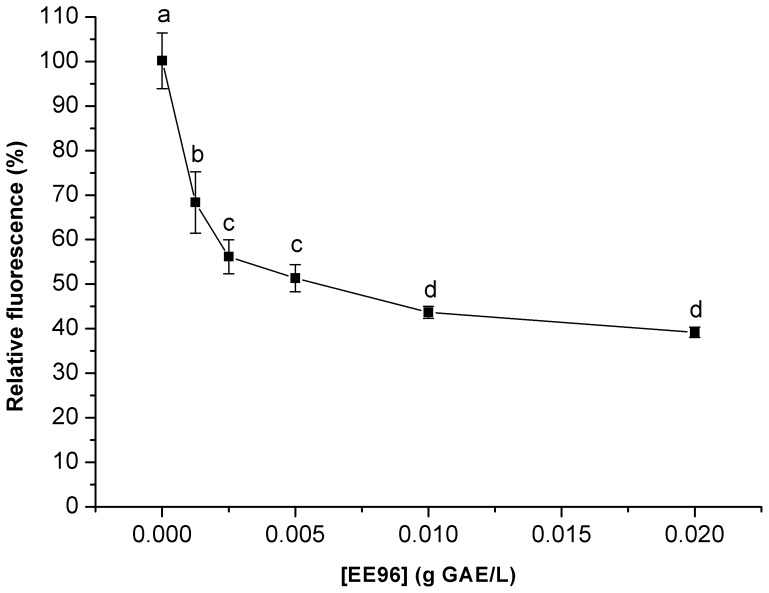
Intracellular oxidation in the yeast *S. cerevisiae* treated with EE96. Data are means (n = 3) and are expressed as fluorescence relative to control. Values followed by the same letter (a–d) are not statistically different (P<0.05), as measured by Duncan’s test.

Therefore, the antioxidative activities of the cleaned propolis EE96 and the eluates (EL30–EL70) that showed free radical scavenging activities were here investigated using an appropriate model organism. These antioxidative activities were determined by measuring intracellular oxidation using the yeast *S. cerevisiae*. The yeast was treated for 2 h with EE96 or the EL30 to EL70 eluates. With EE96 used in the yeast suspensions at concentrations from 0.00125 g GAE/L to 0.02 g GAE/L, there was a dose-dependent decrease in the intracellular oxidation (Fig. 3). This decrease continued to 0.01 g GAE/L EE96, after which it reached a plateau.

**Figure 4:**
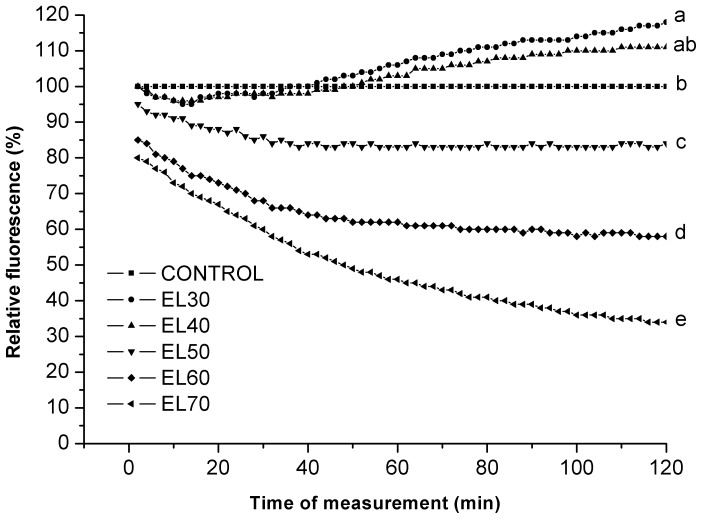
Intracellular oxidation in the yeast *S. cerevisiae* treated with EL30 to EL70 eluates. Data are means (n = 3) and are expressed as fluorescence relative to control. Values followed by the same letter (a–e) are not statistically different (P<0.05), as measured by Duncan’s test.

Next, the yeast suspensions were treated for 2 h with the eluates EL30 to EL70 at 1% (v/v). Preliminary experiments showed that the solvent of 1% (v/v) ethanol has no effects on intracellular oxidation in these yeast suspensions (data not shown). The greatest decreases in the intracellular oxidation were seen when the EL70 eluate was added, followed by eluates EL60 and EL50. In contrast, eluate EL30 showed a trend to an increase in intracellular oxidation, whereas no statistical difference compared to the control was seen for EL40 (Fig. 4). Eluates EL60 and EL70 have the same total phenolic content and free radical scavenging activities (Table 1), although they have different *in vivo* antioxidative activities determined by 2′,7′-DCF. Eluate EL70 decreased the intracellular oxidation by 66%, whereas eluate EL60 decreased it by 42%. The diversity of the phenolic compounds in these eluates would be a reason for this difference. Eluates EL30 and EL50 both have free radical scavenging activity, although they show different effects on yeast intracellular oxidation. In the case of eluate EL50, the intracellular oxidation decreased by 16%, whereas eluate EL30 increased it by 18%. Therefore, these data indicate that in these yeasts, the free radical scavenging activities of these eluates do not always correlate with their antioxidative activities.

As well as the antioxidative activities, insights into other biological activities are also of importance. Therefore, we investigated the cellular metabolic energy and the viability of these treated yeast.

**Figure 5:**
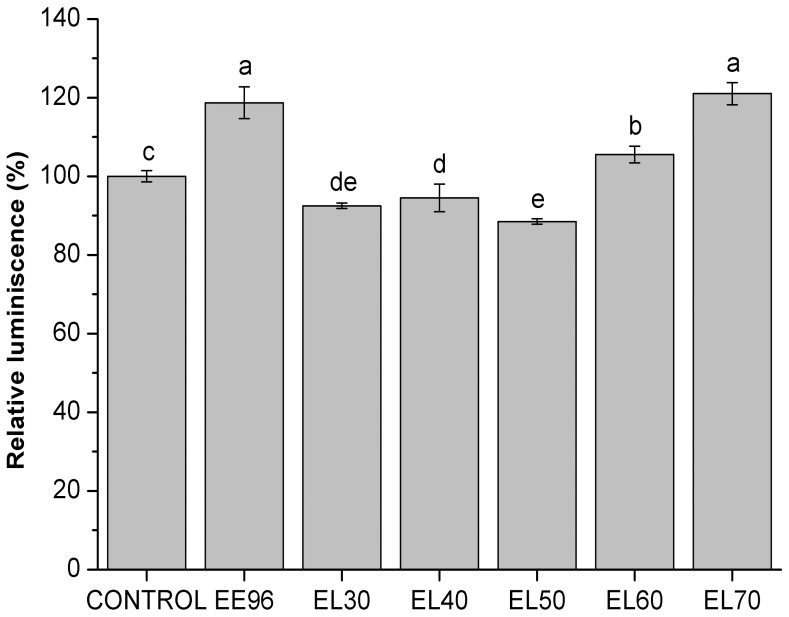
Cellular metabolic energy in the yeast *S. cerevisiae* treated with EE96 and EL30 to EL70 eluates (1%). Data are means ±S.D. (n = 3) and are expressed as luminescence relative to control. Values followed by the same letter (a–e) are not statistically different (P<0.05), as measured by Duncan’s test.

For the cellular metabolic energy, eluates EL60 and EL70 again showed differences compared to the others, as seen in Figure 5. An increase in cellular metabolic energy was seen when eluates EL60 and EL70 were added to the yeast suspensions at the final concentration of 1% (v/v). Despite the same concentration of total phenolics in these two eluates (Table 1), eluate EL70 showed a greater increase in cellular metabolic energy (21%) than seen for EL60 (6%). This difference in effects between these eluates was already seen in the case of intracellular oxidation. With eluates EL30 to EL50, there were small, but significant, decreases in the cellular metabolic energy compared to the control (Fig. 5). As also seen in Figure 5, the cellular metabolic energy increased (19%) in the yeast treated with the cleaned propolis EE96 at the same final concentration of 1% (v/v).

**Figure 6:**
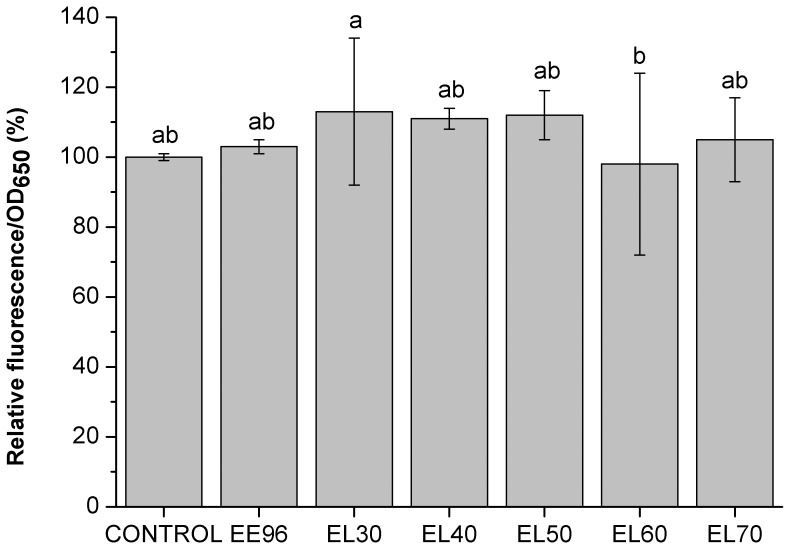
Cell viability of the yeast *S. cerevisiae* treated with EE96 and EL30 to EL70 eluates (1%). Data are means ±S.D. (n = 3), and are expressed as fluorescence normalized to OD_650_ relative to control. Values followed by the same letter (a-b) are not statistically different (P<0.05), as measured by Duncan’s test.

Cell viability was measured as cell-membrane integrity using the nucleic-acid-specific dyes SYTO®9 and propidium iodide in the LIVE/DEAD® Funga Light TM Yeast Viability kits (Molecular Probes). These data showed no significant changes in the viability of the yeast treated with the cleaned propolis EE96 and the EL30 to EL70 eluates at this final concentration of 1% (v/v) (Fig. 6). It was previously demonstrated that Brazilian propolis induces cell death in *S. cerevisiae* [13]. As stated, no such effect was observed in our study, where stationary yeast cells as a model organism were used. We also tested activity of eluate EL70 in the exponential growth phase, where the same effect (decreased intracellular oxidation and no changes in cell viability) as in stationary growth phase was observed (data not shown). Thus, activity of eluate EL70 is growth-phase independent. Therefore, different results might be due to the differences in the composition of propolis and concentration of its phenolic compounds, since it is known that European propolis differ from Brazilian propolis [1].

**Figure 7:**
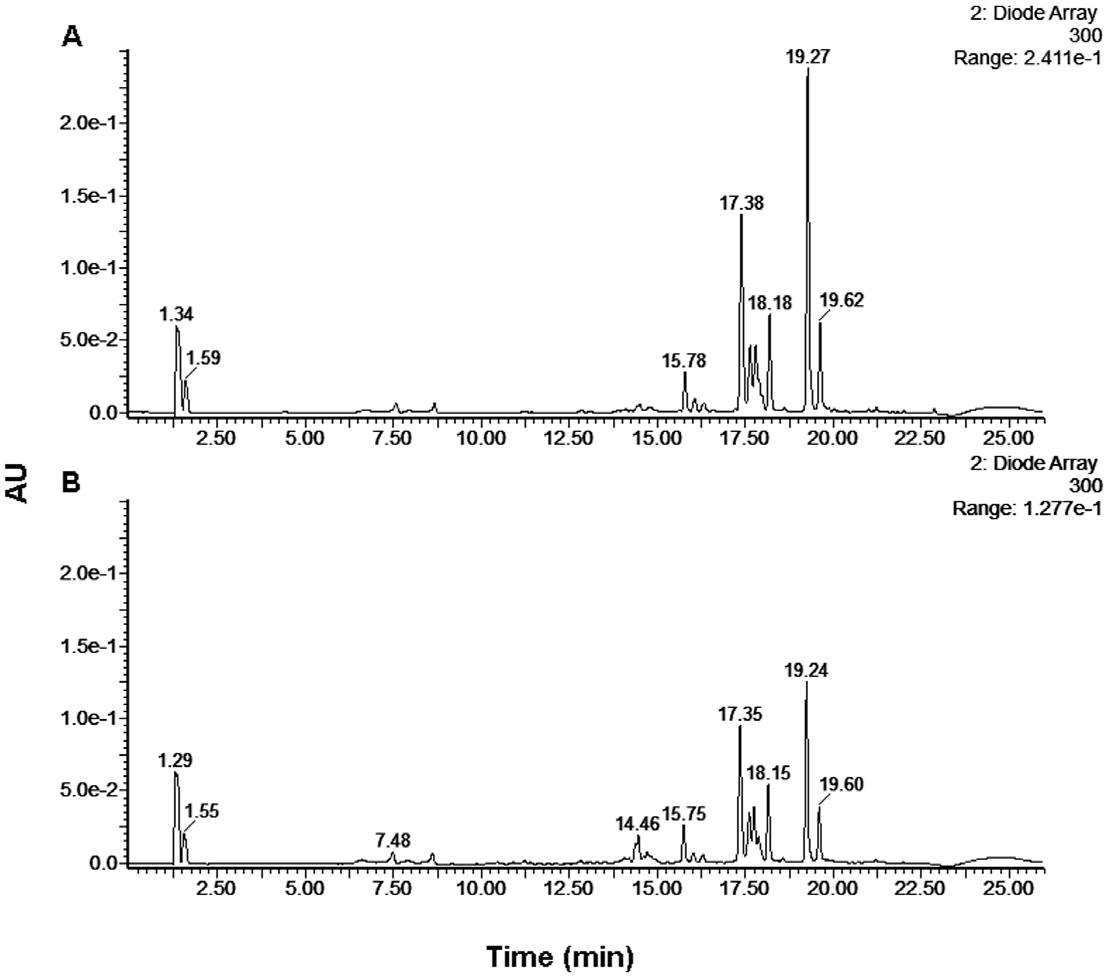
LC-DAD (300 nm) chromatogram of the phenolic compounds in the incubation medium before (A) and after (B) 2 h of exposure of the yeast *S. cerevisiae* to eluate EL70 (1%). AU, arbitrary units.

As the antioxidative activities of phenolic compounds in the cell have been shown to be connected with their cellular uptake [7], the uptake from the particular eluates by these yeasts was also investigated here. The LC-DAD profile of the incubation medium before and after exposure of these yeast to the various eluates was determined. Here, some of the LC-DAD peaks of the eluate EL70 samples were decreased after treatment (Fig. 7: retention times: 17.32±0.05 min; 18.17±0.02 min; 19.26±0.02 min; 19.61±0.01 min), whereas no changes in these LC-DAD profiles were seen before and after the treatments with eluates EL30 to EL50 (data not shown). These decreased LC-DAD peaks in eluate EL70 were also seen in EL60, but to a lesser extent. This might be a reason for the higher antioxidative activity of eluate EL70 compared to eluate EL60 (Fig. 4). Despite no apparent cellular uptake from eluate EL30, it increased intracellular oxidation (Fig. 4). This might be explained in terms of many phenolic compounds being unstable in cell-culture media, whereby they can undergo rapid oxidation, to generate hydrogen peroxide and other ROS [14]–[17]. Among these products, hydrogen peroxide can pass through cell membranes by passive diffusion, which would contribute to an increase in intracellular oxidation [18].

Thus, we have shown initially that there is cellular uptake, a decrease in intracellular oxidation, and an increase in cellular metabolic energy in yeast treated with eluate EL70. This led to further investigation of the individual phenolic compounds in this EL70 fraction of the crude propolis extract. Here we used LC-MS/MS and focussed on the LC-DAD retention times from 17 min to 20 min.

**Figure 8:**
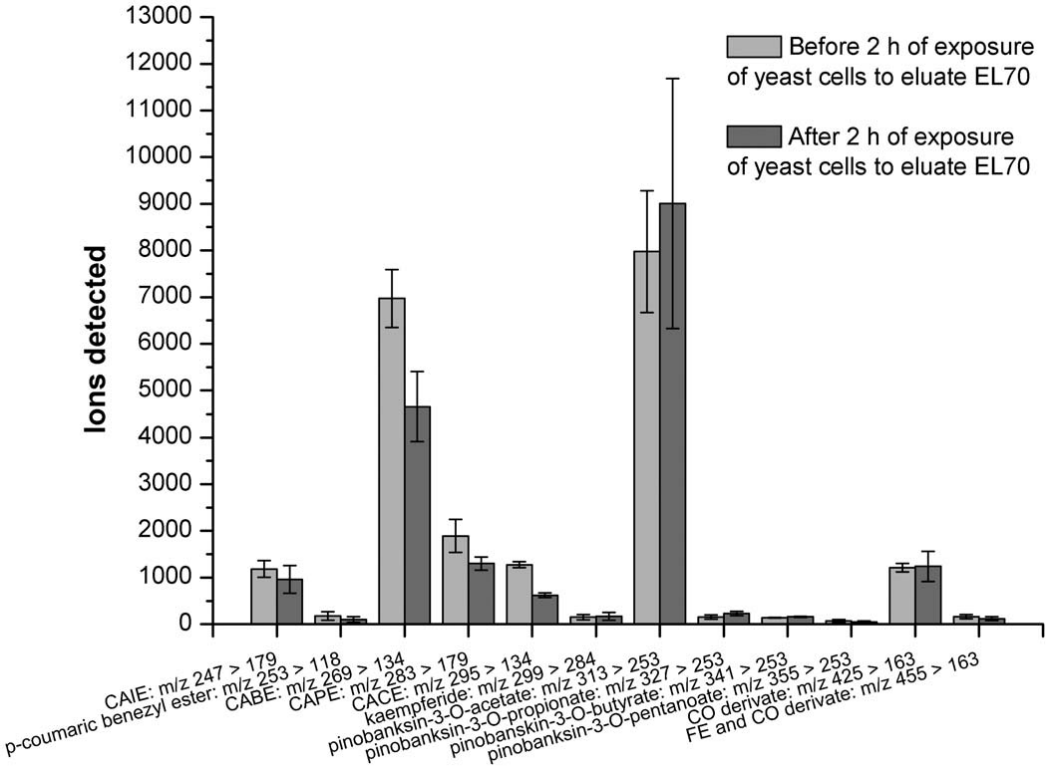
LC-MS/MS of the phenolic compounds in the incubation medium before and after 2 h of exposure of the yeast *S. cerevisiae* to eluate EL70 (1%). Data are means ±S.D. (n = 3), and are expressed as the detected ions. CAIE, caffeic acid isoprenyl ester; CABE, caffeic acid benezyl ester; CAPE, caffeic acid phenethyl ester; CACE, caffeic acid cinnamyl ester; CO, coumaric acid; FE, ferulic acid.

Based on comparison of our m/z of [M-H]^−^ and MS^2^ ions with those described in the literature [19]–[24], we were able to identified the following phenolic compounds: caffeic acid esters (caffeic acid isoprenyl ester, caffeic acid benezyl ester, caffeic acid phenyethyl, caffeic acid cinnamyl ester), p-coumaric benzyl ester, flavonoids (chrysin, pinocembrin, apigenin, kaempferide, rhamnetin), phenolic acid derivates (caffeic acid derivate, coumaric acid derivate, ferulic acid derivate), pinobanksin derivates (pinobanksin-5-methyl-ether, pinobanksin-3-O-acetate, pinobanksin-3-O-propionate, pinobanksin-3-O-butyrate, pinobanksin-3-O-pentanoate) and luteolin-methyl-ether and 3-prenyl-4-(2-methylpropionyl-oxy)-cinnamic acid) (Table 2). This identification of the individual phenolic compounds enabled investigation of their cellular uptake in more detail. The parent/daughter ions of individual phenolic compounds that were detected before and after 2 h of exposure of the yeast to 1% (v/v) eluate EL70 were compared (Fig. 8). Ions from caffeic acid benezyl ester and pinobanskin-3-O-acetate dominated. Significant decreases in the ions detected were observed for caffeic acid benezyl ester, caffeic acid phenethyl ester, caffeic acid cinnamyl ester. Other phenolic compounds that are not shown were identified in eluate EL70, but were only present at trace levels.

**Table 2:** Phenolic compounds identified in eluate EL70 using mass spectrometry detection.

[M-H]^−^ (m/z)	MS^2^ ions (m/z)	Compound	Reference
247	134 179	Caffeic acid isoprenyl ester	[20]
253	143	Chrysin	[19]
253	118	*p*-Coumaric benzyl ester	[21]
255	107 151	Pinocembrin	[20]
269	117 107	Apigenin	[22]
269	134	Caffeic acid benezyl ester	[20]
283	179 135 161	Caffeic acid phenethyl ester	[23]
285	252 138 224 165	Pinobanksin-5-methyl-ether	[19]
285	165 185	Unknown	
285	135 163	CA- and CO-derivate	[19]
295	134	Caffeic acid cinnamyl ester	[20]
299	179 135	CA-derivate	[19]
299	227 255	Luteolin-methyl-ether	[19]
299	284 151 164	Kaempferide	[21]
313	253 107	Pinobanksin-3-O-acetate	[20]
315	271 255	3-Prenyl-4-(2-methylpropionyl-oxy)-cinnamic acid	[21]
315	165 121	Rhamnetin	[24]
327	253	Pinobanksin-3-O-propionate	[20]
341	163 119	CO-derivate	[19]
341	253	Pinobanksin-3-O-butyrate	[20]
355	253	Pinobanksin-3-O-pentanoate	[20]
381	119 135 179 163	CA- and CO-derivate	[19]
404	294	Unknown	
425	163	CO-derivate	[19]
455	163 193	FE- and CO-derivate	[19]
457	179 161 235 295 135	CA-derivate	[19]
471	193 179 235 135 175 161	CA- and FE-derivate	[19]

CA, caffeic acid; CO, coumaric acid; FE, ferulic acid.

To our knowledge, this is the first study where solid-phase extraction has been used for fractionation of phenolic compounds from crude propolis, with the investigation of the various activities of the individual eluates investigated in yeast as a model organism: intracellular oxidation, cellular metabolic energy, and cell viability. Additionally, we have identified the cellular uptake of individual phenolic compounds that may contribute to the antioxidative and cellular metabolic energy effects of propolis in yeast.
